# A meta-synthesis of qualitative literature on female chronic pelvic pain for the development of a core outcome set: a systematic review

**DOI:** 10.1007/s00192-021-04713-1

**Published:** 2021-04-06

**Authors:** Vishalli Ghai, Venkatesh Subramanian, Haider Jan, Ranee Thakar, Stergios K. Doumouchtsis, CHORUS: An International Collaboration for Harmonising Outcomes, Research and Standards in Urogynaecology and Women’s Health

**Affiliations:** 1 Department of Obstetrics and Gynaecology, Epsom & St Helier University Hospitals NHS Trust, Dorking Road, London, KT18 7EG UK; 2 St George’s University of London, Crammer Terrace, London, SW17 0RE UK; 3 Department of Urogynaecology, Croydon University Hospital NHS Trust, London, CR7 7YE UK; 4 Laboratory of Experimental Surgery and Surgical Research N.S. Christeas, Athens University Medical School, Athens, Greece; 5 School of Medicine, American University of the Caribbean, Cupecoy, Sint Maarten

**Keywords:** Core outcome set, Chronic pelvic pain, Meta-synthesis, Qualitative, Systematic review

## Abstract

**Introduction and hypothesis:**

Qualitative research has an increasing role in the development of core outcome sets (COS) adding patient perspectives to the considerations of core outcomes. We aimed to identify priorities of women with experience of chronic pelvic pain (CPP).

**Methods:**

The search strategy was a systematic review of qualitative studies identified from Cochrane Central Register of Controlled Trials (CENTRAL), CINAHL, EMBASE, MEDLINE and PsycInfo databases. Selection criteria were qualitative studies exploring the experience of women with CPP. Two independent researchers extracted data and summarized findings using thematic analysis. A CERQual assessment was performed to assess the confidence of review findings.

**Results:**

We identified pertinent issues affecting women with CPP including the lack of holistic care, influence of psychosocial factors and the impact of pain on quality of life. Five meta-themes central to delivering a patient-centred approach were highlighted: acceptance of pain, quality of life, management of CPP, communication and support. Management of CPP was the most commonly reported meta-theme across seven studies and half of studies reported quality of life, management, communication and support. Quality appraisal of included studies identified only a single study that met all CASP (Critical Appraisal Skills Programme) criteria. There was high confidence in the evidence for acceptance of pain, quality of life and communication meta-themes.

**Conclusion:**

Meta-themes revealed by this review should be considered as a priority and reflected in outcomes reported by future studies evaluating interventions for CPP. In addition, these themes should be considered by clinicians managing women with CPP.

**Supplementary Information:**

The online version contains supplementary material available at 10.1007/s00192-021-04713-1.

## Introduction

Chronic pelvic pain (CPP) is a debilitating condition in severe cases and affects 15% of women worldwide [1]. It is associated with significant long-term morbidity, increased healthcare utilization and socio-economic burden [2]. It is defined as pain lasting > 6 months or recurrent episodes of abdominal/pelvic pain, hypersensitivity or discomfort often accompanied with elimination changes and sexual dysfunction [3].

Qualitative research is underused despite its potential to inform and improve the quality of care of women with CPP [4]. Current standard medical and surgical interventions lack a holistic approach thereby failing to improve pain intensity and quality of life outcomes [5]. The evidence to support such treatments is extrapolated from quantitative research. However, the complexities of pain sensation require research beyond the benefits and harms of an intervention as demonstrated by clinical trials.

There is increasing use and emphasis of qualitative evidence synthesis (QES) within clinical, public health, policy and healthcare systems. A criticism of primary qualitative studies is the lack of generalisability of findings beyond the population studied. However, a QES is the combination and analysis of individual qualitative studies, bringing together multiple perspectives, which may not be represented by a single study [6]. QES seek to develop an understanding of health related behaviours, experiences of illness, evaluation and implementation of complex interventions. Researchers are able to gain a greater understanding of individual’s experiences, views, beliefs and priorities for healthcare [7].

Transparent reporting of QES has emerged as an important area of consideration. Standardized reporting is necessary to facilitate the appropriate use of qualitative evidence into policy and practice. A modified GRADE approach, CERQual (Confidence in the Evidence from Reviews of Qualitative research) provides a systematic and transparent framework assessing how much confidence to place in findings from QES [8]. It consists of four components including: (1) the methodological limitations of the individual qualitative studies contributing to a review finding, (2) the coherence of the review finding, (3) the adequacy of data supporting a review finding and (4) the relevance to the review question of the individual studies contributing to a review finding.

To date, there is no previous QES exploring the experience of women with CPP that has applied a CERQual approach. Earlier QES have been limited, often descriptive and lacking in standardized quality assessment of recommendations [9–12]. In this study, we aimed to perform a QES describing the views and perspectives of women with CPP. We analysed findings using a CERQual approach with a focus on insights that may be useful in the process of establishing core outcome sets (COS) in CPP.

## Materials and methods

This study was registered with the International Prospective Register of Systematic Reviews (PROSPERO), registration number CRD42019141856, and is reported in accordance with the ENTREQ statement guidelines [13].

This study has been performed by a working group of CHORUS, an International Collaboration for Harmonizing Outcomes, Research and Standards in Urogynaecology and Women’s Health (https://i-chorus.org), aiming to address outcome reporting, research quality and research and clinical standards in the areas of urogynaecology, female pelvic medicine and reconstructive surgery [14–16]. This project is part of a wider initiative to develop core outcome sets (COS) in therapeutic interventions for female CPP that has been prospectively registered with the Core Outcome Measures in Effectiveness Trials (COMET initiative), number 981. The development and implementation of a COS will improve the quality of studies, promote greater reporting consistency as well as reduce outcome reporting bias. The proposed qualitative meta-synthesis was conducted in parallel to developing an inventory of reported outcomes and outcome measures in quantitative studies.

### Inclusion and exclusion criteria

We included qualitative studies evaluating the experience of women with CPP that used qualitative methods for data collection and analysis. Mixed method studies were not considered to ensure a homogenous sample. Studies not written in English were excluded.

### Search strategy

Our search strategy was based on the recommendations for identifying qualitative reports [17–19]. A comprehensive literature search was undertaken using MEDLINE, The Cochrane Central Register of Controlled Trials (CENTRAL), EMBASE, CINAHL and PsycInfo databases. Searches were performed from database inception to December 2019 using the following search terms: “qualitative research”, “qualitative study” “interview”, “focus group” and “chronic pelvic pain”.

### Study selection

Potentially eligible studies were screened using titles and abstracts. Full text articles were retrieved for abstracts meeting the inclusion criteria or in cases when information in the abstract was incomplete or unclear. Reference lists of included articles were hand-searched to identify any additional studies. Full text articles were reviewed by two independent researchers and discrepancies regarding suitability for inclusion were resolved by discussion with a senior author (SKD). A flow chart is included to demonstrate the search and study inclusion process (Appendix S1).

### Quality assessment of included studies

Two independent researchers assessed the methodological quality of all included studies using an adaptation of the Critical Appraisals Skills Programme (CASP) tool (https://www.casp-uk.net) for qualitative studies. This tool consists of ten questions assessing the rigour, credibility and relevance of the study. Disagreements were resolved by consensus or discussion with a third senior researcher (SKD). No studies were omitted on the basis of quality assessment as this may exclude valuable perspectives in this field. Furthermore, this process enabled weaknesses in studies to be highlighted, thereby allowing findings to be interpreted more effectively whilst taking these limitations into account. In the data synthesis section, we described how we integrated and utilized study quality assessments to analyse review findings.

### Data extraction

Two independent researchers extracted study characteristics from eligible studies using a piloted data extraction form. Study characteristics included author details, year of publication, journal, objectives, study design, number of participants, method of data collection and analysis.

### Data analysis and synthesis

Thematic synthesis was used to analyse qualitative data from included studies. Data included direct quotations from participants describing their experience of CPP. Synthesis was conducted in three stages as described by Thomas et al. [20]. Initially, narratives from study participants were coded line by line to identify similarities. In the second stage, codes and data were grouped together to create descriptive themes. In the final stage of thematic synthesis, distinct analytical themes were defined. The analysis was conducted collaboratively by two researchers using Microsoft Excel (2018). Discrepancies were resolved through discussion with a third senior author (SKD).

### Assessing the confidence of findings

The confidence of each review finding was assessed using the CERqual tool. The CERqual approach consists of four components: (1) methodological limitations of individual studies contributing to the review finding, (2) relevance of findings to the review question, (3) coherence and (4) adequacy of data. The methodological limitations of individual studies contributing to review findings were assessed using the CASP tool. The relevance of individual studies to the review question were assessed based on the extent to which review findings are applicable to the context (perspective, population, phenomena of interest, setting) as specified in the review question. The coherence of each review finding was assessed by exploring whether there is support found in underlying data from contributing studies. The adequacy of data was assessed by considering the number of studies and participants as well as the richness of data contributing to review findings. These four components were assessed and described having none, minor, moderate or serious concerns. Based on an overall assessment, the confidence in the evidence for each review finding was scored as high, moderate or low.

### Patient and public involvement

There has been no patient involvement in this review as this study is a meta-synthesis of existing research. However, during the development of a COS, stages such as Delphi surveys and consensus meetings will include patient participation and involvement.

## Results

This systematic review was reported in accordance with the ENTREQ statement guidelines to enhance transparency in reporting QES [13]. Results are presented using flow charts and summary tables of study characteristics, qualitative findings and confidence assessments.

The literature search was conducted in December 2019 and identified 1239 titles and abstracts. We screened 1019 titles and abstracts after the exclusion of 220 duplicate records (Appendix S1). We finally included eight studies (references of these studies appear in Appendix S2) with a total of 201 female participants, ranging between 19 to 70 years of age (Table S1). The most common data collection methods were: semi-structured interviews (5/8 studies, 62.5%), open-ended interviews (2/8 studies, 25%) and written stories (1/8 studies, 12.5%). Data analysis was performed using four methods and included: phenomenology (3/8 studies, 37.5%), constant comparison (2/8 studies, 25%), grounded theory (2/8 studies, 25%) and thematic analysis (1/8 studies, 12.5%).

The quality appraisal of included studies scored a median value of 8 (out of a maximum of 10, interquartile range 7.75–9) (Table S2). There was only a single study (1/8 studies, 12.5%) that met all CASP criteria. The majority of studies (7/8 studies, 87.5%) performed poorly when examining the researchers’ own role and potential bias.

**Fig. 1:**
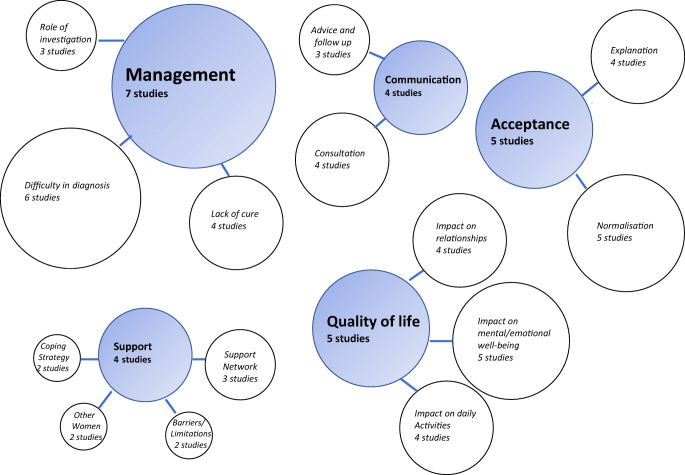
Diagrammatic representation of the relationship between meta-themes and sub-themes

Thematic synthesis of qualitative data from included studies identified 91 codes. Similar codes were grouped together into 15 sub-themes and further organized into five meta-themes (Table S3). Meta-themes included: acceptance of pain, quality of life, management of chronic pelvic pain, communication with health professionals and support (Fig. 1 and Table S3). The meta-themes and sub-themes highlighted by our analysis were described by women suffering from CPP and are included in Appendix S3. The CERqual approach was used to establish confidence in the evidence to relation to each meta-theme (Table S4).

### Acceptance of pain

“Acceptance of pain” was a meta-theme present in over of half studies (5/8 studies, 62.5%). This meta-theme refers to how participants describe, understand and rationalize the pain they experience. “Acceptance of pain” comprised two thematic categories: “normalisation of symptoms” and “underlying causes of pain” (Table S3). Women normalized their symptoms by attributing them to physiological processes (menstruation, intercourse) and conceptualized their condition as a “woman’s problem” as well as denying the extent of symptoms by pretending to act stoic through pressure to continue as “normal”. Participants proposed explanations to illustrate their understanding and probable causes of their pain. This appeared to be beneficial as it facilitated a process of acceptance among women in comparison to the anxiety and distress observed in instances when no cause was given for the pain. Explanations were often mechanical in nature and cited damage sustained during childbirth as well as stressors experienced during life events such as bereavement and abortion (Appendix S3).

There was high confidence in this evidence for this meta-theme. Five studies contributed to this meta-theme with minor methodological limitations (Table S4). Studies were conducted across three countries and 135 participants were recruited from various sources: a database acquired during a prevalence study and secondary care and tertiary care outpatient clinics.

### Quality of life

This meta-theme was present in almost two-thirds of studies (5/8 studies, 62.5%) and describes the individual and wider detrimental impact of CPP. Three subthemes, “impact of pain on activities of daily living”, “impact of pain on mental/emotional well-being” and “impact of pain on relationships” contributed to the “quality of life” meta-theme (Table S3). Women describe a sense of loss compared to their once former normal self as their physical symptoms prevent them from fulfilling previous roles and responsibilities. The impact of CPP is widespread, affecting activities of daily living, performance at work, participation in social activities and maintaining healthy relationships. The disruptive nature of CPP poses a particular problem and is a frequent cause of anxiety among women with CPP. Women described a loss of control due to the unpredictable nature of CPP that contributes to the psychological morbidity (poor self-esteem, feelings of worthlessness) associated with such a disorder (Appendix S3).

There was high confidence in this evidence for this meta-theme. Five studies contributed to this meta-theme with minor methodological limitations (Table S4). Studies were conducted across three countries and 109 participants were recruited from a database acquired during a prevalence study in secondary and tertiary care hospitals.

### Management of chronic pelvic pain

This meta-theme encapsulates the frustrating journey of many women searching for a diagnosis and treatment for CPP. Over 85% of studies (7/8 studies) reported this meta-theme and comprised three subthemes including: “difficulty in reaching or a lack of a diagnosis”, “lack of cure” and “the role of diagnostic tests” (Table S3). Women emphasized the importance of a diagnosis to exclude sinister pathology such as cancer, which was the source of anxiety among many women. There was a clear emphasis on objective evidence obtained from investigations by both participants and health professionals. A clear lack of knowledge was evident as investigations may not help diagnose CPP or the underlying cause itself. Negative investigations appeared to be damaging for women as these failed to legitimize their experience and attach a diagnosis to their symptoms. Furthermore, in light of negative investigations women were misdiagnosed with psychiatric disorders. A lack of diagnosis or cure resulted in women feeling alienated and disempowered with limited avenues of medical and social support to alleviate their CPP (Appendix S3).

There was moderate confidence in the evidence for this meta-theme. Seven studies with moderate methodological limitations contributed to this meta-theme (Table S4). Studies were conducted across three countries and 150 participants were recruited from a database acquired during a prevalence study, newspaper advertisements, and secondary and tertiary care hospitals.

### Communication

This meta-theme was present in half of studies and refers to the interaction with health professionals and specifically focuses on how this influences the experience of women with CPP. Two subthemes, “consultation” and “advice and follow-up” contributed to the “communication” meta-theme (Table S3). Women described negative encounters in which health professionals appeared to be rushing through consultations, lacking in empathy and failing to recognize the severity or impact of symptoms. In some cases, this resulted in women withdrawing and disengaging from medical services and failing to seek advice. In comparison, a positive encounter eluded to a health professional that established a rapport by acknowledging a patient's experience as well as symptoms and utilized visual aids to assist clear communication. Furthermore, women expressed a lack of follow-up after procedures and advice following a diagnosis (Appendix S3).

There was high confidence in this evidence for this meta-theme. Four studies contributed to this meta-theme with minor methodological limitations (Table S4). Studies were conducted across one country and 87 participants were recruited from newspaper advertisements, secondary and tertiary care hospitals.

### Support

“Support” was a meta-theme present in 50% of studies and consisted of four subthematic categories: “other women with chronic pelvic pain”, “support network”, “barriers and limitations to support” and “personal traits and coping strategies” (Table S3). This meta-theme encapsulates the struggle of women with chronic pain and the strategies they employ to function productively and effectively as possible. These include sharing experiences with other women with CPP. Such exchanges reduce the feelings of isolation and allow a direct comparison, which form attitudes to reduce anxiety and uncertainty. Family and friends were also important sources of emotional and practical support. However, there appeared to be a delicate balance involved in using support from family and friends. Women were acutely aware of being a burden and in some cases this prevented them from disclosing the full extent of their illness. Additionally, women encountered difficulties when communicating their pain due to embarrassment, stoicism and disbelief from individuals within their support network. The development of coping strategies improved the ability to cope with pain and involved the identification of exacerbating factors as well as distraction techniques to shift focus from the illness allowing women to relax mentally and physically. It was also evident that personal traits such as self-motivation and self-belief were crucial components of resilience and maintaining self-worth. These contributed to effective coping strategies. Women reported a lack of long-term and sustained support to match the chronicity of their condition (Appendix S3).

There was moderate confidence in the evidence for this meta-theme. Four studies contributed to this meta-theme with minor methodological limitations (Table S4). Studies were conducted across two countries and 69 participants were recruited from secondary and tertiary care hospitals.

## Discussion

### Main findings

We identified five meta-themes: acceptance of pain, quality of life, management of chronic pelvic pain, communication and support. Management of chronic pelvic pain was the most common reported meta-theme across seven studies and half of studies reported quality of life, management, communication and support. The most common method of data collection was semi-structured interviews and data analysis was conducted most frequently using a phenomenology approach. Quality appraisal of included studies only identified a single study that met all CASP criteria. The majority of studies performed poorly when examining the researchers’ own role and potential bias. There was high confidence in the evidence for acceptance of pain, quality of life and communication meta-themes. There was moderate confidence in evidence for management of CPP and support meta-themes.

### Strengths and limitations

To our knowledge, this is the first meta-synthesis of female CPP that uses the CERQual approach to systematically and transparently assess the confidence placed in review findings. We used robust and reproducible methods including independent analysis from two researchers to minimize bias arising from data selection, collection and analysis. This methodology has been successfully applied in previous studies and is an established study design [14]. Furthermore, to improve the scientific rigour of this review, a quality assessment of included studies was performed using the CASP tool. By implementing a CERQual approach to grade evidence, we have increased the quality and application of our findings to inform decision-making processes. We adopted an interpretative approach to data synthesis as this facilitated the reinterpretation of evidence to produce an in-depth analysis rather than superficially aggregating and summarizing existing qualitative evidence. We developed overarching constructs that “go beyond” the findings articulated within individual primary studies.

This study has limitations. We limited our search to qualitative studies, potentially missing information arising from mixed method and quantitative studies. Only studies in English were included to minimize issues arising from translating terms and the subsequent definition and classifications into sub-themes and meta-themes. Studies were from developed countries and the experiences described may not be generalized to other healthcare systems. Included studies were published between 1996 to 2008; advances in diagnostics and treatments may not be reflected in these experiences. However, this indicates a lack of primary research in CPP and is a priority for future research.

### Interpretation

In this meta-synthesis, we examined the experience of women with CPP and identified insights to inform a future COS. In an era of patient-centred care, qualitative research has an increasing role in promoting patients’ interests as well improving the quality and standard of care. Previous COS in other areas of obstetrics and gynaecology have successfully utilized and incorporated findings from primary qualitative research. Findings identified additional outcomes which were missed by previous systematic reviews of clinical studies [21]. Although our study is a meta-synthesis, it explores generically and provides a broad perspective of what matters most to women with CPP including health and social themes.

Findings from this meta-synthesis are a valuable contribution to the development of a meaningful COS in CPP. Authors of clinical trials often select and report outcomes without patient/user consultation or involvement. Furthermore, studies investigating interventions for CPP may include outcomes such as “satisfaction”, which is a poor indicator of what a positive or negative experience entails [22]. Pertinent issues related to management of conditions may be overlooked and not reflective of patient priorities. For example, a systematic review of reported outcomes in therapeutic interventions in CPP demonstrated that quality of life was only reported by half of trials [23]. However, our study indicated that poor quality of life was a prevalent and reoccurring theme for women suffering with CPP. There is a difference in the prominence of outcomes given by clinical trials and qualitative literature. For this reason, it is imperative that qualitative studies such as ours are used in conjunction and complement findings from systematic reviews to generate outcomes for consideration and inclusion into Delphi surveys, consensus meetings and a final COS.

Additionally, we will use women’s own narratives, collected by this study, to help describe and label themes/domains using appropriate language. This will ensure that subsequent Delphi surveys are accessible and can easily be understood by key stakeholders participating in the survey such as patients. Insights from this study have facilitated a deeper understanding of which themes/domains matter most to women and why. Determining the scope of the themes/domains is vital to ensure accurate and relevant outcomes are forwarded in the COS development process as well as selection of suitable outcome measures, for example, the inclusion and impact of psychosocial factors to maximize treatment outcomes.

Our findings identified areas of concerns such as interactions with health professionals and medical services and the lack of holistic treatment including the recognition and influence of psychosocial factors. However, the experience of women with CPP is not unique but shared across a number of chronic pain conditions. Previous meta-syntheses exploring chronic pain condition syndromes have also alluded to delays in diagnosis, failure to recognize the individual and wider impact of pain symptoms among health professionals as well as a lack of interventions supporting women and their families [12, 24, 25]. Such feedback allows identification of areas of improvement whilst ensuring patients’ views and opinions contributes to service development. The deeper understanding of patient priorities resulting from qualitative research may not have been possible using quantitative research methodology.

In this review, women with CPP described their negative experiences of healthcare services and highlighted the lack of individualized care, a holistic approach and continuity of care. These insights can be utilized to frame the overarching theme of future guidelines and inform a model of care women want and health providers want to provide. Future clinical priorities should focus on developing a multi-disciplinary and patient-centred approach. At present, psychological and behavioural therapies are neglected despite the overwhelming evidence supporting their use in reducing pain intensity. The recognition and correction of pain behaviours and concomitant mood disorders are necessary to optimize potential benefits derived from conventional medical/surgical treatments [11, 26]. Targeted psychological therapies such as cognitive behavioural therapy have proven effective at reducing pain catastrophization [11]. Additionally, the persistent and pervasive nature of chronic pain requires patients to make adjustments to learn to live with their disease by implementing effective coping strategies. Inclusion of behavioural interventions that promote protective psychological factors and coping mechanisms should be considered in multifaceted treatment programmes. Active pain coping (APC) and acceptance and commitment therapy (ACT) are beneficial in cultivating traits such as resilience and self-efficacy [27, 28].

This review evaluated the methodological quality of individual qualitative studies using the CASP checklist. However, only a single study met all CASP criteria. For qualitative evidence to be incorporated into practice and policy, it is necessary that future qualitative studies implement robust methodology. Authors of qualitative studies should pay particular attention to the researchers as a source of potential bias. Efforts such as pilot studies in which researchers trial their proposed methods are helpful in identifying researcher biases [29]. The confidence of our review findings ranged from moderate to high confidence despite methodological concerns. Although methodological limitations may lower our confidence in review findings, they may not necessarily lead to downgrading of overall confidence in review findings, as this was assessed alongside the other three CERQual components [30].

Future research priorities should concentrate on developing and implementing a COS in CPP. Although we have conducted a meta-synthesis and a systematic review of clinical trials, we feel there is benefit in conducting a primary qualitative study. This review indicated moderate confidence in evidence pertaining to support and management of CPP meta-themes. A primary qualitative study will facilitate the exploration of these areas and specifically ascertain what women expect and need from CPP healthcare provision. Furthermore, previous groups developing COS noted that additional outcomes were identified using various research methodologies [21]. Findings from this meta-synthesis will be used to inform the agenda for semi-structured interviews and focus groups. Additionally, secondary analysis studies are in progress to investigate the discordance between key stakeholder groups as well as compare outcomes generated by this meta-synthesis and our previous systematic review. These findings will be useful in advising and understanding methodological considerations in the development of COS.

## Conclusion

Findings from this meta-synthesis will ensure that patient priorities, as key stakeholders, are reflected in outcomes reported by future studies evaluating interventions for chronic pelvic pain.


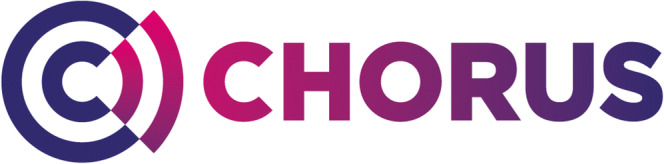


## Supplementary Information

ESM 1 (DOC 31 kb)

ESM 2 (DOCX 15 kb)

ESM 3 (DOCX 39 kb)

ESM 4 (DOCX 15 kb)

ESM 5 (DOCX 15 kb)

ESM 6 (DOCX 15 kb)

ESM 7 (DOCX 14 kb)
