# Supplementary material for: A meta-synthesis of qualitative literature on female chronic pelvic pain for the development of a core outcome set: a systematic review
Source: Int Urogynecol J. 2021 Apr 6;32(5):1187–94. doi: 10.1007/s00192-021-04713-1 (PMC8139940; doi:10.1007/s00192-021-04713-1)
Supplement: Supplementary file 1 — (DOC 31 kb) [file 192_2021_4713_MOESM1_ESM.doc]

**Appendix S1. PRISMA 2009 Flow Diagram**

**Screening**

**Included**

**Eligibility**

**Identification**

Records identified through database searching
(n = 1,239)

Additional records identified through other sources
(n = 0)

Records after duplicates removed
(n = 220)

Records screened
(n = 1,019)

Records excluded
(n =1,004)

Full-text articles assessed for eligibility
(n = 15)

Full-text articles excluded, with reasons
(n =6 )

Not qualitative data collection or analysis (3)

Mixed methods (1)

Not relevant (1)

No raw qualitative data (1)

Systematic review (1)

Studies included in qualitative synthesis (metasynthesis)
(n =8)
