# Supplementary material for: A meta-synthesis of qualitative literature on female chronic pelvic pain for the development of a core outcome set: a systematic review
Source: Int Urogynecol J. 2021 Apr 6;32(5):1187–94. doi: 10.1007/s00192-021-04713-1 (PMC8139940; doi:10.1007/s00192-021-04713-1)
Supplement: Supplementary file 2 — (DOCX 15 kb) [file 192_2021_4713_MOESM2_ESM.docx]

**Appendix S2. References of included studies**

Grace VM, MacBride-Stewart S. “Women get this”: gendered meanings of chronic pelvic pain. Health (London). 2007 Jan;11(1):47–67.

Grace VM, MacBride-Stewart S. “How to say it”: women's descriptions of pelvic pain. Women Health. 2008;46(4):81–98. DOI: 10.1300/J013v46n04_05

McGowan L, Luker K, Creed F, Chew-Graham CA. How do you explain a pain that can't be seen?: the narratives of women with chronic pelvic pain and their disengagement with the diagnostic cycle. Br J Health Psychol. John Wiley & Sons, Ltd; 2007 May;12(Pt 2):261–74.

Moore J, Ziebland S, Kennedy S. "People sometimes react funny if they‘re not told enough": women’s views about the risks of diagnostic laparoscopy. Health Expect. John Wiley & Sons, Ltd; 2002 Dec;5(4):302–9.

Price J, Farmer G, Harris J, Hope T, Kennedy S, Mayou R. Attitudes of women with chronic pelvic pain to the gynaecological consultation: a qualitative study. BJOG. John Wiley & Sons, Ltd; 2006 Apr;113(4):446–52.

Savidge CJ, Slade P, Stewart P, Li TC. Women's Perspectives on their Experiences of Chronic Pelvic Pain and Medical Care. J Health Psychol. SAGE Publications Sage CA: Thousand Oaks, CA; 1998 Jan;3(1):103–16.

Warwick R, Joseph S, Cordle C, Ashworth P. Social support for women with chronic pelvic pain: what is helpful from whom?, Psychology & Health. 2004.19:1, 117-134, DOI: [10.1080/08870440310001613482](https://doi.org/10.1080/08870440310001613482)

Zadinsky JK, Boyle JS. Experiences of women with chronic pelvic pain. Health Care Women Int. 4 ed. Taylor & Francis Group; 1996 May;17(3):223–32.
