# Supplementary material for: A meta-synthesis of qualitative literature on female chronic pelvic pain for the development of a core outcome set: a systematic review
Source: Int Urogynecol J. 2021 Apr 6;32(5):1187–94. doi: 10.1007/s00192-021-04713-1 (PMC8139940; doi:10.1007/s00192-021-04713-1)
Supplement: Supplementary file 3 — (DOCX 39 kb) [file 192_2021_4713_MOESM3_ESM.docx]

**Appendix S3. Quotations from the articles included illustrating different themes (A-E)**

| 1. Acceptance of pain |  |  |
| --- | --- | --- |
| Study | **Quotations** | **Authors’ Interpretation** |
| Warwick et al 2004 | “Every woman suffers, don’t worry about it.” | “Acceptance of pain. There is a belief that the pain only affects women and is conceptualised as a woman’s problem.” |
| Savidge et al 1998 | “He just said he couldn’t find anything, that he didn’t know what was causing the pain. I couldn’t understand it because I thought well something’s got to be causing it. Surely there must be something there, the pain can’t be there for nothing, can it? There must be some reason why I’m in pain ... I felt very strange about it. I mean did he think I was pretending or something?” | “Expression of concern and anxiety as no cause of pain found. An explanation regarding pain is important to women in terms of validating their experience of illness and symptoms. Women felt like frauds as no cause found to legitimise their pain.” |
| Price et al 2006 | “I didn’t want to go down the drugs scene, I wanted an answer, I wanted to know what it was, not just to feel that I was masking it.” | “Indicating that it is important to know the cause of pain rather than receiving only a cure.” |
| Grace et al 2007 | “I just thought all pain down in this area was connected with periods and that was that. And it was sort of a woman’s lot that you had to accept it type of thing.” | “Acceptance of pain due to being a woman and relating it normal physiological processes” |
| Grace et al 2007 | “Yeah, I just thought maybe a lot of people got it after having sex and I didn’t think it was, yeah, a big deal … I just thought, yeah, everyone gets pain and that.” | “Chronic pain also has far reaching consequences such as impact on sex. Trivialising symptoms, process of normalisation, lacking in knowledge about what it normal and abnormal. Some women who experienced pain during sex attributed pain due to mechanical causes such as a childbirth retroverted uterus, size of their partners’ penis.” |
| Grace et al 2008 | “Feeling like I’m, you know, in more pain than some of the patients and having to slap a smile on my face when I really did not feel like smiling (. . .) and just not concentrating (. . .)” | “Having to pretend to be stoic and tolerate pain. Pressure and obligation felt by women to manage and carry on at home and work.” |
| Grace et al 2007 | “You know, when you’ve had babies they [internal organs] tend to get a bit of a battering … oh yes, yeah, everything gets sort of beaten up a little bit.” | “Some women attributed pain due to mechanical damage caused by  childbearing and childbirth. It opposes idea that birth is natural versus the internal damage caused by seemingly normal process. Women often expressed an acceptance that childbirth may cause damage. Women articulated mechanical based explanations for their pain such as strain and stretching of birth, effect of stitches and muscles cut during caesarean section.” |
| Savidge et al 1998 | “It was due to stress I think. About four years ago, when I was 16 my boyfriend died of leukaemia and I had a lot of stress around that time. With being only 16 it were a bit of a shock, and a loss ... the time the pain started was the time it hit me most. Obviously I was distressed after he died, but a couple of years ago it really hit me hard and I couldn’t get him out of my mind and it started coming back and that sort of thing.” | “Women felt that events other than physiological processes such as could also be responsible for their pain. These include stressors such as bereavement and abortion.” |
| Warwick et al 2004 | “All I want is for them to diagnose it so I can deal with it. . .If you know what the monster is you can deal with it. If you don’t know what it is your brain just makes a bigger one.” | “Women described difficult with having no label attached for pain. Presents as difficulty as cannot legitimise condition as well finding support.” |

| 1. Quality of life |  |  |
| --- | --- | --- |
| Study | **Quotations** | **Authors’ Interpretation** |
| Savidge et al 1998 | “It’s affected everything, my social life, everything. I’ve changed from being outgoing, playing badminton, swimming, walking ... I used to walk for miles, we were campers and we stopped it all. because I were petrified of something happening out on the moors.” | “Illustrates the far reaching consequences of pain on activities of daily living and social activities. The unpredictability and severity of pain prevents women from participating in activities they once enjoyed. Also reference to “healthy self” before chronic pelvic pain.” |
| Grace et al 2008 | “The pain was just absolutely terrible, especially for two or three days it was, you know affected me so badly that I couldn’t, sometimes you know, continue at work, I’d have to come home.” | “Impact of pain on work and accounts for poor performance and inability to function.” |
| Grace et al 2008 | “Oh it just makes me stop sometimes and it makes me moody, and, [. . .] I don’t like being like that with my children…” | “Emotional impact of pain on relationships and fulfil personal roles such as being a mother. Also there is guilt displayed as not been able to fulfil such roles” |
| Warwick et al 2004 | “Because I’m in this pain it’s him I’m going to pick on all the time.” | “Detrimental effect of pain affecting relationship with partners. Pain causing women to be angry and irritable.” |
| Savidge et al 1998 | “When I get the pain bad it’s very serious to me. I honestly think sometimes I’m going to just die, it’s so bad, I just can’t explain how bad it is.” | “Personal impact of pain causing psychological distress. Pain seen as frightening experience. Some women described weight loss and sleep disturbance. Cyclical nature and unpredictability of pain episode are a further source of anxiety among women.” |
| Warwick et al 2004 | “I know that the sex issue is big on his part. I can block off but obviously if there’s pain there you can’t do it. He gets very frustrated about that.” | “Pain affecting sexual relationships, frustration displayed by partners.” |
| Savidge et al 1998 | “I worry that I might not be able to have children one day, that niggles at me. I suppose it goes through any woman’s mind if they have pain down there.” | “Women experience anxiety due to location of pelvic pain and proximity to reproductive organs may mean difficulties conceiving children in the future.” |
| Zadinsky et al 1996 | “Your self-esteem goes down the drain. I don't feel very confident about myself anymore because I don't know what's going on inside my own body. I don't have any control over it.” | “Loss of control over life and worthlessness due to unpredictable nature of pain. This has a negative impact on how individual views “one self”.” |

| 1. Management |  |  |
| --- | --- | --- |
| Study | **Quotations** | **Authors’ Interpretation** |
| Price et al 2006 | “A lot of people in my family have died of cancer and, it was crazy, but there’s things that go on in the back of your mind, ovarian cancer and all those sort of things that it could have been, but no-one was giving you any explanation…” | “Need for an explanation of pain as women experience anxiety pain may be caused by sinister pathology such as cancer.” |
| McGowan et al 2007 | “How do you explain a pain that can’t be seen?” | “Indicates frustrating search for a diagnosis and the emphasis placed on objective evidence of pain and therefore confirmation using investigations. If no physical evidence or signs are found, this can lead to alienation and the continued search for an explanation. Indicates lack of understanding regarding pathophysiology of chronic pelvic pain.” |
| Price et al 2006 | “You feel very despondent, you don’t know who to listen to, this person tells you something so you go and see someone else and they tell you something different. So you’re left not knowing.” | “Loss of hope and frustration as uncertainty about diagnosis or a lack of diagnosis.” |
| Price et al 2006 | “She sent me to see a specialist at the hospital and they couldn’t find anything wrong . she was very abrupt and a pain in the bum really (laughs). I didn’t like her at all and she said that I think you should just see a psychiatrist . she said she couldn’t do anything*.*” | “As chronic pelvic pain may be associated with a lack of physical signs, women may be dismissed and misdiagnosed with a psychological illness. Women are labelled as neurotic, anxious or depressed and continue to experience chronic pelvic pain.” |
| Savidge et al 1998 | “Well I weren’t sure what it could of been but I know there’s got to be an explanation for pain, there’s no smoke without fire, there’s got to be some explanation you don’t just get pain for no reason*.*” | “Lack of understanding regarding chronic pelvic pain pathophysiology. There is a desire for an explanation of pain, disbelief at negative investigations.” |
| Price et al 2006 | “I was quite anxious that he wasn’t going to find anything and that I’d been making up all this, worrying about nothing really, and . [after the positive result] a sense of relief struck me when he found out it [endometriosis] had all come back again.” | “Women experience relief if a cause of their pain is found. Positive investigation validates symptoms and legitimises their experience of pain as well as helps women understand their pain.” |
| McGowan et al 2007 | “Gynaecologist No. 2 booked me for a scan and a laparoscopy, he was delighted to inform me that there was nothing of any relevance inside. How could I be in so much pain with nothing causing it? I was devastated.” | “Indicates emphasis placed on investigations by doctors and patients. The manner in which the negative results are communicated to women is crucial in their acceptance and understanding of their pain.” |
| Moore et al 2002 | “I accept there’s an accepted risk in all things, I, in the foremost of my mind was that I was going to find out what was wrong with me and what was causing the pain, and that overrode anything else that was maybe associated risk-wise with the operation.” | “Attitude to the risk of interventions such as a laparoscopy. Women felt the information provided by the surgery such as the cause of pain outweighed the risks of the surgery.” |
| Savidge al 1998 | “I mean it’s very hard to explain to somebody. When I actually went to see him (the doctor) I didn’t have the pain which has happened on a lot of occasions and so it was very difficult to pinpoint where the pain was ... he gave me the impression that he just didn’t believe me, that I was wasting his time.” | “Intermittent and unpredictability of pain pose a challenge for diagnosis.” |
| McGowan et al 2007 | “I’m not sure if anything can help with this condition, a cure would be good or some recognition that this condition does exist for some women and no we don’t have “Munchausens syndrome” or are attention seekers, but a genuine pain and would like tohave better things to do than be dismissed and left in limbo, I’m no further on all these years later of a doctor telling me what I have or how he his going to treat me than the very first time I went to see him.” | “Indicates the lack of recognition and cure associated with chronic pelvic pain. Women are devalued, disempowered and are left with limited avenues of medical and social support.” |

| 1. Communication |  |  |
| --- | --- | --- |
| Study | **Quotations** | **Authors’ interpretation** |
| Price et al 2006 | “I wouldn’t say that I wasn’t believed, but I was made to feel that I was exaggerating or making more of it than was really there, and that basically just get on with it and live with it. I wouldn’t say that they didn’t believe me but they certainly made me feel like I was exaggerating.” | “Perception that not taken seriously or believed by health professionals leads to a negative encounter.” |
| Warwick et al 2004 | “Before we start let me just tell you that there’s nothing wrong with you’ and then proceeded to give me a big speech about why women worry too much about their symptoms and women under 30 don’t have any serious problems.” | “Lack of recognition of chronic pelvic pain by health professionals. They are influenced by their own pre-conceived ideas which may impact their management chronic pelvic pain. Responses indicate a lack of understanding and empathy.” |
| Price et al 2006 | “It was very impersonal. It sort of felt like if you went to MacDonald’s and went through a fast food place like that. I mean because they have so many people to see they just want to get you through as quickly as possible.” | “Doctors reported as cold and unfriendly, these were explained by time pressures faced during consultation and number of patients waiting to be seen.” |
| Savidge et al 2008 | “He asks you awkward questions and in fact they’re a bit embarrassing some of the questions, I didn’t know how to answer them. I mean when you go about ’ought personal like that, I think it should be a lady doctor, because they examine you and you have to take your clothes off ... and your legs are wide open and they’re shaking like mad.” | “Women may have a preferences for a female doctor during a consultation. This is due to the difficulties and embarrassment that may arise from explaining where and when the pain arises as well as during ta physical examination.” |
| McGowan et al 2007 | “With this, I was so upset and embarrassed that I did not return to the doctor for a long time but “put up with it” – assuming, that as I had been told everything was normal, it was just something to be endured.” | “Negative consultations with health professionals such as which lack of empathy and recognition of symptoms result in women withdrawing and disengage from seeking attention.” |
| Warwick et al 2004 | “This was the first time that somebody had said, ‘It wasn’t your fault, it shouldn’t have happened. Yes all these terrible things that happened to you, did happen.’ I knew then that it wasn’t in my head. It was almost as if I was able to put it to bed.” | “Positive consultations are those in which health professionals acknowledge the pain experienced by women. It is important part of validation and acceptance for the women as wll as knowing they are not responsible. Positive consultations can help women cope with pain.” |
| Price et al 2006 | “She sat down and let me explain everything and I said to her, and she didn’t just say about the medical side of things it was about my personal, it was how it affects me personally which I thought was really nice and she was like very sympathetic and understanding and she was saying oh I’m ever so sorry we haven’t been able to help you before.” | “Positive consultation resulting from an empathetic and supportive approach. Involves recognising acknowledging the impact of pain on the individual.” |
| Savidge et al 1998 | “She was great, she listened and she explained and she was everything a gynae doctor should be-her manner and her approach ... she sits down on the bed in hospital and talks to you and doesn’t just stand hovering and she didn’t make me feel intimidated so I’d forget what to ask her.” | “Importance of body language during communication with patients as well as giving time for an explanation and opportunity for questions. This resulted in a positive and satisfactory encounter as the woman feels listened to as opposed to dismissed.” |
| Warwick et al 2004 | “I think the doctors who’ve taken time to try and explain how things work, if you like, try to say, ‘This is the problem you’ve got’, and draw diagrams and, ‘This is what’s happened, and this is why we’re doing this’. To actually give a rationale and information about what’s going on, that’s helpful.” | “Clear communication and use of visual aids can assist communication between doctors and women.” |
| Price et al 2006 | “You’re never really told [the outcome of the laparoscopy] .  you’re told that you’ve got endometriosis but that’s sort of.  again you’re not really told whether it’s bad, whether it’s  mild, whether it’s . you’re not told anything like that,  and you’re told while you’re still asleep, while you’re still  coming round from the anaesthetic.” | “Importance of post-operative debriefing and ensure all patients concerns and questions are addressed” |
| Warwick et al 2004 | “Told me I got ‘Endometriosis, It’s to do with the womb’, and that was it. . .I was told I got a disease that I didn’t know anything about. I thought I was dying, to be honest.” | “Need for information and long-term management are necessary for patients to cope and treat disease” |

| 1. Support |  |  |
| --- | --- | --- |
| Study | **Quotations** | **Authors’ interpretation** |
| Warwick et al 2004 | “When somebody your age gets the same, similar kinds of pain and has similar kinds of restrictions, it is helpful to think that I’m not really that weird.” | “Women valued support from other women with chronic pelvic pain. Women are able to relate to other women and share experiences. Also helps reduce isolation as well allow comparison to form attitudes and help cope with uncertainty and anxiety resulting from such an illness” |
| Savidge et al 1998 | “Well I’d go to your own doctor and shout very loud and say you know, ’listen to me’, and I’d ask to see somebody at the hospital ...and don’t take no rubbish from him ... to stand her ground with him and say ’listen to me’, and don’t shut up until they do ... and don’t be fobbed off by them.” | “Women may find advice from other women with chronic pelvic pain helpful. Illustrates challenges in seeking medical help and advice.” |
| Zadinsky et al 1996 | “There were a few times when I had given up, and I said I just couldn't go on; and he would always come to me and say yes you can, you can do it, you're strong. So I went on.” | “Partners and friends are an important source of social support for women with chronic pelvic pain. This may be in the form of emotional or practical support” |
| Warwick et al 2004 | “Oh God you’ve always got something wrong, you’re always ill.” | “Women are aware that they may be perceived as a burden on their support network. They fear exhausting the support of friends and family. This may prevent them disclosing the full extent of their illness or masking the problem.” |
| Warwick et al 2004 | “...gets very frustrated that he can’t help me, he cannot take the pain away, there is not a lot he can do, bar get me a hot water bottle, and he gets so frustrated with me and that kind of turns into anger.” | “Anger and frustration expressed by people in their support network. This is due to the feeling of inadequacy and ineffective at reducing pain and suffering of women with chronic pelvic pain.” |
| Warwick et al 2004 | “My dad tends to get involved when he has a strong idea about something...he is really quite forceful if he has got an idea...so he can be quite unhelpful and my mum, when they really do try to push things on me.” | “Highlights danger of support provided by social network becoming ineffective as it undermines a woman’s autonomy. Women may perceive such well-intentioned support as interfering rather than helpful.” |
| Warwick et al 2004 | “My problem has been cutting off and keeping it in, you know not being able to express. . .I said ‘this is it, bowels problem, always opening my bowels when I go to the toilet, the whole thing’s a mess, its just too embarrassing’. . .He (partner) will go, ‘I don’t want to know that’.” | “Identifies challenges in accessing support due to difficulties in communicating to family and friends. There is a need to empower women so they feel comfortable to discuss their pain as well overcoming the stigma of embarrassing topics.” |
| Warwick et al 2004 | “At times when I get a stabbing pain, he doesn’t know when that’s happened, and I might just not be communicating about it.” | “The lack of physical signs of chronic pelvic pain makes it difficult to communicate their pain to family and friends and therefore access support. This may be accompanied by disbelief from friends and family therefore leading to isolation. Stoicism may also hinder communication.” |
| Warwick et al 2004 | “It was useful, very useful to have the written information, because I think when I was told how severe the condition was . it upset me quite a lot and probably I wasn’t taking everything in that they were telling me . at least I had the written information to take home with me and I could digest it.” | “Indicates lack of written information related to chronic pelvic pain.” |
| Warwick et al 2004 | “When I was in hospital last week, they were absolutely brilliant but now I’m home it’s as though nothing has happened.” | “Women expressed need for sustained and enduring long-term support to match the chronicity of their condition.” |
| Savidge et al 2004 | “The G.P. said it can be very much stress related ... and when I get stressed or run down, then I notice it flares up so I now know what it is and can adapt round it when I know it’s starting to flare.” | “Identifying of exacerbating factors can lead to an improved ability to cope with pain by developing strategies to prevent and overcome pain. Coping strategies may also include distraction techniques to shift the focus from illness and allow women to relax physically and mentally.” |
| Zadinksy et al 1996 | “I have no reason to feel bad and not to want to get up and not to want to do things, but, you know, I doubt myself and say, are you actually feeling bad? You know you're not senile or crazy, but are you just playing it up worse than what it is? I honestly know in my head I'm not.” | “Personal traits such as self-motivation and self-belief are crucial components of resilience as well as maintaining self-worth. These can contribute to effective coping strategies.” |
