# Supplementary material for: A meta-synthesis of qualitative literature on female chronic pelvic pain for the development of a core outcome set: a systematic review
Source: Int Urogynecol J. 2021 Apr 6;32(5):1187–94. doi: 10.1007/s00192-021-04713-1 (PMC8139940; doi:10.1007/s00192-021-04713-1)
Supplement: Supplementary file 4 — (DOCX 15 kb) [file 192_2021_4713_MOESM4_ESM.docx]

**Table S1. Study characteristics**

| **Author; year** | **Study title** | **Journal** | **Objectives** | **Country** | **Source** | **Number of participants (age)** | **Method of data collection and data analysis** |
| --- | --- | --- | --- | --- | --- | --- | --- |
| **Grace et al, 2007** | 'Women get this': gendered meanings of chronic pelvic pain. | Health: An international journal for the social study of health, illness and medicine | To explore the meanings of female gender and pain with regards to the experience  of women with CPP | New Zealand | Prevalence survey | 40  (22-50) | Open-ended interviews  Phenomenology |
| **Grace et al, 2008** | "How to say it": women's descriptions of pelvic pain. | Women & Health | To investigate women's descriptions of CPP and comparison with descriptors of  pain used in a pain assessment tool | New Zealand | Prevalence survey | 40  (22-50) | Open-ended interviews  Phenomenology |
| **McGowan et al, 2007** | How do you explain a pain that can't be seen?: the narratives of women with chronic pelvic pain and their  disengagement with the diagnostic cycle. | British Journal of Health Psychology | To explore the processes which lead to disengagement of women from health services despite continuing to have symptoms and to understand the psychological processes that affect these women | United Kingdom | Newspaper article | 32  (21-50) | Written stories  Phenomenology |
| **Moore J et al, 2002** | 'People sometimes react funny if they're not told enough': Women's views about the risks of diagnostic  laparoscopy. | Health Expectations | To explore women's views of risks and how much information is appropriate to provide about three specific risks: death, major complication and no cause of CPP found at laparoscopy | United Kingdom | Hospital waiting list for laparoscopy | 20  (not specified) | Semi-structured interviews  Constant comparison |
| **Price J et al, 2006** | Attitudes of women with chronic pelvic pain to the gynaecological consultation: A qualitative study. | British Journal of Obstetrics and Gynaecology | To describe the attitudes of women with CPP attending the gynaecology clinic to their consultations and to determine the ways in which their health care can be improved | United Kingdom | Four outpatient gynaecology clinics in district and teaching hospitals | 26  (18-70) | Semi-structured interviews  Grounded theory |
| **Savidge et al, 1998** | Women's perspectives on their experiences of chronic pelvic pain and medical care. | Journal of Health Psychology | To understand the experience of women with CPP and their perception of the care they receive after a negative laparoscopy | United Kingdom | Negative diagnostic laparoscopy for chronic pelvic pain | 21  (21-60) | Semi-structured interviews  Dey’s method |
| **Warwick et al, 2004** | Social support for women with chronic pelvic pain: What is helpful from whom? | Psychology and Health | To examine the impact of social support in women with CPP | United Kingdom | Consultants in two hospitals | 8  (21-61) | Semi-structured interviews  Thematic analysis |
| **Zadinsky et al, 1996** | Experiences of women with chronic pelvic pain. | Health Care for Women International | To describe self-care strategies that women with CPP use to function in their social and  cultural roles | United States of America | Regional medical centre | 14  (19-52) | Semi-structured interviews  Constant comparison |
