# Supplementary material for: A meta-synthesis of qualitative literature on female chronic pelvic pain for the development of a core outcome set: a systematic review
Source: Int Urogynecol J. 2021 Apr 6;32(5):1187–94. doi: 10.1007/s00192-021-04713-1 (PMC8139940; doi:10.1007/s00192-021-04713-1)
Supplement: Supplementary file 5 — (DOCX 15 kb) [file 192_2021_4713_MOESM5_ESM.docx]

**Table S2. CASP summary, by criterion**

| **Criteria** | **Grace et al, 2007** | **Grace et al, 2008** | **McGowan et al, 2007** | **Moore et al, 2002** | **Price et al, 2006** | **Savidge et al, 1998** | **Warwick et al, 2004** | **Zadinsky et al, 1996** |
| --- | --- | --- | --- | --- | --- | --- | --- | --- |
| Explicitly stated aims and objectives | ✓ | ✓ | ✓ | ✓ | ✓ | ✓ | ✓ | ✓ |
| Appropriate use of qualitative methods | ✓ | ✓ | ✓ | ✓ | ✓ | ✓ | ✓ | ✓ |
| Justification for methods used | ✓ | ✓ | ✓ | ✓ | ✓ | ✓ | ✓ | ✓ |
| Appropriate method of recruitment, description of recruitment | ✓ | ✓ | ✓ | Can’t tell | ✓ | ✓ | Can’t tell | ✓ |
| Description of setting, methods of data collection, form of data recorded | ✓ | ✓ | Can’t tell | Can’t tell | ✓ | ✓ | ✓ | ✓ |
| Critical examination of researchers’ own role and potential bias | Can’t tell | Can’t tell | No | No | ✓ | Can’t tell | No | Can’t tell |
| Evidence of ethical approval | ✓ | ✓ | ✓ | ✓ | ✓ | No | ✓ | ✓ |
| Description of method used, adequate and rigorous in-depth data analysis | Can’t tell | ✓ | ✓ | ✓ | ✓ | ✓ | ✓ | ✓ |
| Clear statement of findings, discussion of evidence, credibility and validity of findings | ✓ | ✓ | ✓ | ✓ | ✓ | ✓ | ✓ | ✓ |
| Contribution to existing knowledge, transferrable to clinical practice, improvement of patient care | Can’t tell | ✓ | ✓ | ✓ | ✓ | ✓ | ✓ | ✓ |
| **Total scores** | 7 | 9 | 8 | 7 | 10 | 8 | 8 | 9 |
