# Supplementary material for: A meta-synthesis of qualitative literature on female chronic pelvic pain for the development of a core outcome set: a systematic review
Source: Int Urogynecol J. 2021 Apr 6;32(5):1187–94. doi: 10.1007/s00192-021-04713-1 (PMC8139940; doi:10.1007/s00192-021-04713-1)
Supplement: Supplementary file 6 — (DOCX 15 kb) [file 192_2021_4713_MOESM6_ESM.docx]

**Table S3. Meta-themes and sub-themes identified in studies**

| **Meta-themes;**  ***sub-themes*** | **Grace et al, 2007** | **Grace et al, 2008** | **McGowan et al, 2007** | **Moore et al, 2002** | **Price et al, 2006** | **Savidge et al, 1998** | **Warwick et al, 2004** | **Zadinsky et al, 1996** |
| --- | --- | --- | --- | --- | --- | --- | --- | --- |
| **Acceptance of pain** | **X** | **X** |  |  | **X** | **X** | **X** |  |
| Normalisation of pain | ***** | ***** |  |  | ***** | ***** | ***** |  |
| Causes/explanation of pain | ***** |  |  |  | ***** | ***** | ***** |  |
|  |  |  |  |  |  |  |  |  |
| **Quality of life** |  | **X** |  |  | **X** | **X** | **X** | **X** |
| Impact of pain on daily activities |  | ***** |  |  |  | ***** | ***** | ***** |
| Impact of pain on emotional/mental well-being |  | ***** |  |  | ***** | ***** | ***** | ***** |
| Impact of pain on relationships |  | ***** |  |  |  | ***** | ***** | ***** |
|  |  |  |  |  |  |  |  |  |
| **Management** | **X** |  | **X** | **X** | **X** | **X** | **X** | **X** |
| Difficulty in reaching or lack of diagnosis | ***** |  | ***** |  | ***** | ***** | ***** | ***** |
| Lack of cure |  |  | ***** |  | ***** | ***** |  | ***** |
| Role of diagnostic tests |  |  | ***** | ***** |  | ***** |  |  |
|  |  |  |  |  |  |  |  |  |
| **Communication** |  |  | **X** |  | **X** | **X** | **X** |  |
| Consultation |  |  | ***** |  | ***** | ***** | ***** |  |
| Advice and follow up |  |  |  |  | ***** | ***** | ***** |  |
|  |  |  |  |  |  |  |  |  |
| **Support** |  |  |  |  | **X** | **X** | **X** | **X** |
| Other women with chronic pelvic pain |  |  |  |  |  | ***** | ***** |  |
| Support network |  |  |  |  |  | ***** | ***** | ***** |
| Barriers and limitation to support |  |  |  |  | ***** |  | ***** |  |
| strategies/ personal traits contributing to coping strategy |  |  |  |  |  | ***** |  | ***** |
