# Supplementary material for: A meta-synthesis of qualitative literature on female chronic pelvic pain for the development of a core outcome set: a systematic review
Source: Int Urogynecol J. 2021 Apr 6;32(5):1187–94. doi: 10.1007/s00192-021-04713-1 (PMC8139940; doi:10.1007/s00192-021-04713-1)
Supplement: Supplementary file 7 — (DOCX 14 kb) [file 192_2021_4713_MOESM7_ESM.docx]

**Table S4. CERQual qualitative evidence profile**

| **Meta-theme** | **Studies contributing to meta-theme** | **Assessment of methodological limitations** | **Assessment of relevance** | **Assessment of coherence** | **Assessment of adequacy** | **Overall CERQual assessment of confidence** | **Explanation of judgement** |
| --- | --- | --- | --- | --- | --- | --- | --- |
|  |  |  |  |  |  |  |  |
| **Acceptance of pain** | Grace et al 2007,  Grace et al 2008,  Price et al 2006,  Savidge et al 1998,  Warwick et al 2004 | Minor methodological limitations (three studies with minor, one study with moderate and one study with no methodological limitations) | Minor concerns about relevance  (three countries represented in studies) | Minor concerns about coherence (theme consistent across five studies) | Minor concerns about adequacy (theme richly described across three studies) | **High confidence** | This finding was graded as high confidence because of minor concerns regarding methodological, relevance, coherence and adequacy limitations |
|  |  |  |  |  |  |  |  |
| **Quality of life** | Grace et al 2008,  Price et al 2006,  Savidge et al 1998,  Warwick et al 2004,  Zadinsky et al, 1996 | Minor methodological limitations (four studies with minor and one study with no methodological limitations) | Minor concerns about relevance (three countries represented in studies) | Minor concerns about coherence (theme consistent across five studies) | Minor concerns about adequacy (theme richly described across three studies) | **High confidence** | This finding was graded as high confidence because of minor concerns regarding methodological, relevance, coherence and adequacy limitations |
|  |  |  |  |  |  |  |  |
| **Management** | Grace et al 2007,  McGowan et al 2007  Moore et al 2002,  Price et al 2006,  Savidge et al, 1998  Warwick et al, 2004  Zadinsky et al, 1996 | Moderate methodological limitations (two studies with moderate, four studies with minor and one study with no methodological limitations) | Minor concerns about relevance (three countries represented in studies) | Minor concerns about coherence (theme consistent across seven studies) | Moderate concerns about adequacy (theme rich described across three studies) | **Moderate confidence** | This finding was graded as moderate confidence because of minor concerns regarding relevance and coherence and moderate concerns regarding methodological limitations and adequacy |
|  |  |  |  |  |  |  |  |
| **Communication** | McGowan et al 2007  Price et al 2006,  Savidge et al, 1998  Warwick et al, 2004 | Minor methodological limitations (three studies with minor methodological limitations and one study with no methodological limitations) | Minor concerns about relevance (three countries represented in studies) | Minor concerns about coherence (theme consistent across four studies) | Minor concerns about adequacy (theme richly described across three studies) | **High confidence** | This finding was graded as high confidence because of minor concerns regarding methodological, relevance, coherence and adequacy limitations |
|  |  |  |  |  |  |  |  |
| **Support** | Price et al 2006,  Savidge et al, 1998  Warwick et al, 2004  Zadinsky et al, 1996 | Minor methodological limitations (three studies with minor and one study with no methodological limitations) | Minor concerns about relevance (three countries represented in studies) | Minor concerns about coherence (theme consistent across three studies) | Moderate concerns about adequacy (theme richly described across two studies) | **Moderate confidence** | This finding was graded as moderate confidence because of minor concerns regarding methodological limitations, relevance, coherence and moderate concerns regarding adequacy. |
